# Clinicopathological Factors Associated With Level V Cervical Lymph Node Metastasis in Oral Squamous Cell Carcinoma: A Prospective Observational Study

**DOI:** 10.7759/cureus.112598

**Published:** 2026-07-13

**Authors:** Nagendra Parvataneni, Ishfaq A Gilkar, Kiran Kumar Devarakonda, Ulhas Paga, Sushmita P., Nuha Saleem, Birupaksha Biswas, Sunil Kumar, Renuka Kattimani, Sharjeel Khan

**Affiliations:** 1 Surgical Oncology, Krishna Institute of Medical Sciences Hospital, Secunderabad, IND; 2 Surgical Oncology, Sher-i-Kashmir Institute of Medical Sciences, Srinagar, IND; 3 Pathology, West Bengal University of Health Sciences, Kolkata, IND; 4 General Surgery, All India Institute of Medical Sciences, Bibinagar, Bibinagar, IND; 5 Ophthalmology, ESIC Medical College and Hospital, Kalnoor, IND; 6 Forensic Medicine, N.K.P. Salve Institute of Medical Sciences and Research Centre, Nagpur, IND

**Keywords:** extracapsular spread, lymphovascular invasion, nodal metastasis, oral squamous cell carcinoma, perineural invasion

## Abstract

Background

Oral squamous cell carcinoma (OSCC) is the most common malignant tumor of the oral cavity. Metastasis to level V cervical lymph nodes is relatively uncommon but is generally associated with advanced disease and more extensive cervical nodal involvement. Identifying clinicopathological factors associated with level V lymph node metastasis may assist clinicians in surgical planning and pathological risk assessment. Therefore, this study aimed to evaluate the association between selected clinicopathological factors and pathological level V cervical lymph node metastasis in patients with OSCC.

Methods

A prospective observational study was conducted at Krishna Institute of Medical Sciences, Secunderabad, Telangana, India, between November 2022 and March 2024. A total of 120 patients with histopathologically confirmed OSCC who underwent definitive surgical treatment were included. Clinical and pathological variables evaluated included tumor size, tumor site, lymphovascular invasion (LVI), perineural invasion (PNI), tumor stage, and extranodal extension (ENE). All cervical lymph nodes dissected during surgery, including those from level V, were submitted separately for histopathological examination. Descriptive statistics were used to summarize the data, while the chi-square test or Fisher's exact test was used to evaluate associations between categorical variables using SPSS (SPSS Statistics for Windows, version 17; SPSS Inc., Chicago, IL).

Results

In the advanced tumor stage, the presence of LVI, PNI, and ENE was significantly associated with pathological level V lymph node metastasis. Level V metastasis was more frequently observed among patients with advanced cervical nodal disease and adverse histopathological characteristics.

Conclusion

Level V cervical lymph node metastasis was uncommon but was predominantly observed in patients with advanced clinicopathological disease characteristics. Larger tumor size, advanced tumor stage, LVI, PNI, ENE, and more extensive cervical nodal involvement were significantly associated with pathological level V lymph node metastasis. These findings may assist clinicians during preoperative assessment and surgical planning; however, they should be interpreted as associations rather than independent predictive factors. Further multicenter studies are required to validate these findings.

## Introduction

Oral cancer is a major public health concern worldwide and remains one of the most common malignancies of the head and neck, with a relatively high prevalence in many regions [1]. The oral cavity comprises several anatomical sites, including the tongue, gingiva, hard palate, lips, and buccal mucosa, each of which demonstrates distinct patterns of malignant transformation [2]. Oral squamous cell carcinoma (OSCC) accounts for approximately 90-95% of all oral malignancies. Early diagnosis and appropriate treatment of OSCC are essential for improving patient outcomes and reducing disease-related mortality [3]. Despite advances in diagnosis and treatment, the heterogeneous biological behavior and variable clinical outcomes of OSCC continue to present significant challenges in patient management [4].

The oral cavity is richly supplied by an extensive network of blood vessels and lymphatic channels, facilitating both local tumor invasion and dissemination of malignant cells to regional lymph nodes [5]. Regional lymph node metastasis is a well-established prognostic indicator in OSCC, as its presence significantly influences treatment planning and overall survival (OS) [6]. Indeed, lymph node metastasis is considered one of the most important prognostic factors in patients with OSCC and is associated with substantially poorer survival outcomes than in patients without nodal involvement [7]. The OS rate among patients with OSCC and lymph node metastasis ranges from 20% to 36% at five years, whereas patients without lymph node metastasis have reported five-year OS rates ranging from 63% to 86% [8]. These marked differences highlight the importance of identifying clinicopathological factors associated with lymph node metastasis to facilitate early risk stratification and optimize treatment strategies [9].

Risk assessment for lymph node metastasis is primarily based on the clinical and histopathological characteristics of the primary tumor, including tumor size, degree of differentiation, pattern of invasion, and host immune response [10]. Although these clinicopathological characteristics provide valuable insight into the metastatic potential of OSCC, their association with cervical lymph node metastasis may vary among patients. Consequently, there remains a need to further evaluate clinicopathological factors associated with level V cervical lymph node metastasis, which is relatively uncommon and often encountered in patients with advanced disease [11].

Level V lymph nodes, located within the posterior triangle of the neck, represent an important site of lymphatic drainage in patients with OSCC. Although metastasis to level V lymph nodes is relatively uncommon, its presence is generally associated with advanced disease, aggressive tumor behavior, and poorer prognosis [12]. Anatomically, level V lymph nodes are subdivided into level Va and level Vb based on their relationship to the transverse cervical artery. Level Va nodes are located superior to the transverse cervical artery, whereas level Vb nodes lie inferior to this landmark. Accurate assessment of level V lymph node involvement is therefore important for disease staging, prognostic evaluation, and surgical planning in patients with advanced OSCC [13].

The current standard of care for patients with OSCC and cervical lymph node involvement is neck dissection, which involves the surgical removal of lymph nodes from selected cervical levels according to the primary tumor site and the extent of lymphatic spread [14]. The principal surgical approaches include radical neck dissection, modified radical neck dissection, and selective neck dissection, each differing in the extent of lymph node removal and preservation of adjacent non-lymphatic structures, such as the spinal accessory nerve, internal jugular vein, and sternocleidomastoid muscle [15].

Although neck dissection remains a cornerstone of OSCC management, the decision to perform the procedure depends on multiple clinical and pathological factors, including tumor characteristics, the patient's overall health status, and the anticipated risks associated with surgery [16]. Advances in imaging modalities, including ultrasonography, computed tomography (CT), and magnetic resonance imaging (MRI), have improved the detection of cervical lymph node metastases, including involvement of level V nodes. However, accurately identifying patients at increased risk of level V lymph node metastasis before surgery remains challenging [17]. Therefore, the present study was undertaken to evaluate the association between selected clinicopathological factors and pathological level V lymph node metastasis in patients with OSCC. The findings may improve understanding of factors associated with level V nodal involvement and contribute to risk assessment and surgical decision-making in appropriately selected patients.

## Materials and methods

Study design and setting

This prospective observational study was conducted in the Department of Surgical Oncology at Krishna Institute of Medical Sciences, Secunderabad, Telangana, India, between November 2022 and March 2024 after obtaining approval from the Institutional Ethics Committee (Approval No. KIMS/ECBMHR/2022/43-06). The study was designed to evaluate the association between selected clinicopathological factors and level V cervical lymph node metastasis in patients with OSCC.

Selection Criteria

The study included patients with histopathologically confirmed OSCC of the oral cavity who were eligible for definitive surgical treatment and whose primary tumor was confined to the oral cavity. All patients underwent surgical management at Krishna Institute of Medical Sciences, and the diagnosis was confirmed by histopathological examination.

Patients with non-squamous cell malignancies, recurrent tumors, previous radiotherapy or chemotherapy, prior surgery for the primary lesion or neck performed outside the institute, recurrent or residual disease, and synchronous or duplicate primary tumors were excluded.

Sample Size

The sample size was determined with reference to previously published observational studies evaluating clinicopathological factors associated with cervical lymph node metastasis in patients with OSCC that included comparable study populations and outcome measures [9,12,13]. Consecutive sampling was employed, and all eligible patients presenting during the study period who fulfilled the inclusion criteria were recruited. Based on the anticipated patient load at the study center and the feasibility of recruitment during the study period, a total of 120 patients were enrolled.

Data collection and study variables

All patients underwent a comprehensive preoperative evaluation, including detailed history taking, complete oral cavity examination, indirect laryngoscopic examination, bilateral cervical lymph node examination, and general physical examination. Histopathological confirmation of OSCC was established by preoperative biopsy.

Orthopantomography (OPG) was performed in patients with early-stage disease to evaluate mandibular involvement. Patients with large primary tumors, trismus, retromolar trigone involvement, or suspected mandibular invasion underwent CT or MRI for further assessment. The choice of imaging modality was based on clinical presentation and the anticipated extent of disease. Cervical lymph node status was evaluated using ultrasonography. Tumors were staged according to the eighth edition of the American Joint Committee on Cancer (AJCC) Tumor, Node, Metastasis (TNM) staging system.

**Figure 1:**
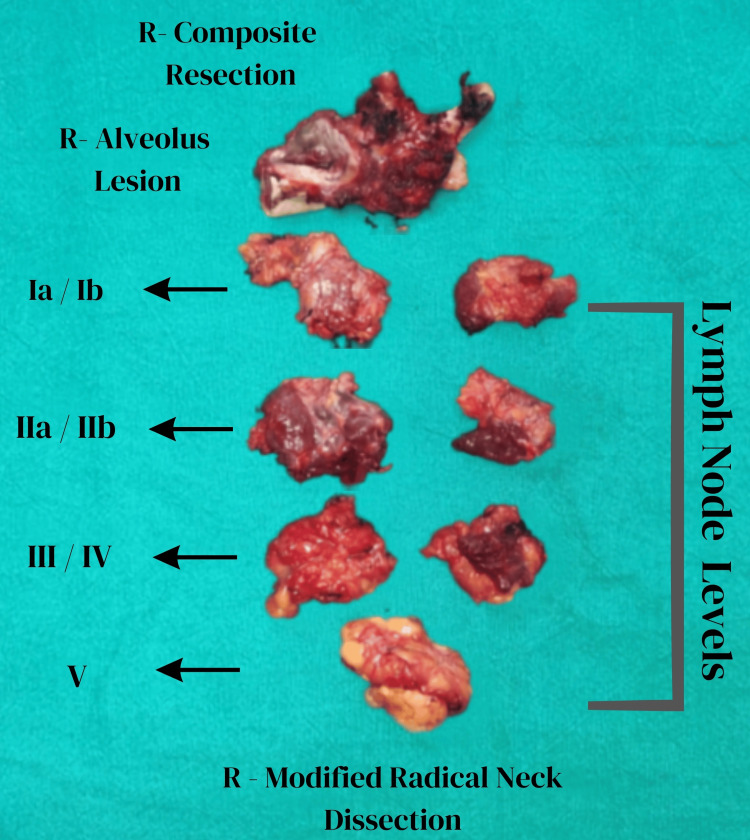
Wide local excision of the right alveolar lesion with right hemimandibulectomy and right modified radical neck dissection specimens Cervical lymph nodes from levels IA, IB, IIA, IIB, III, IV, and V were submitted separately for histopathological examination. R Composite Resection = right composite resection; R Alveolar Lesion = right alveolar lesion; R Modified Radical Neck Dissection = right modified radical neck dissection.

**Figure 2:**
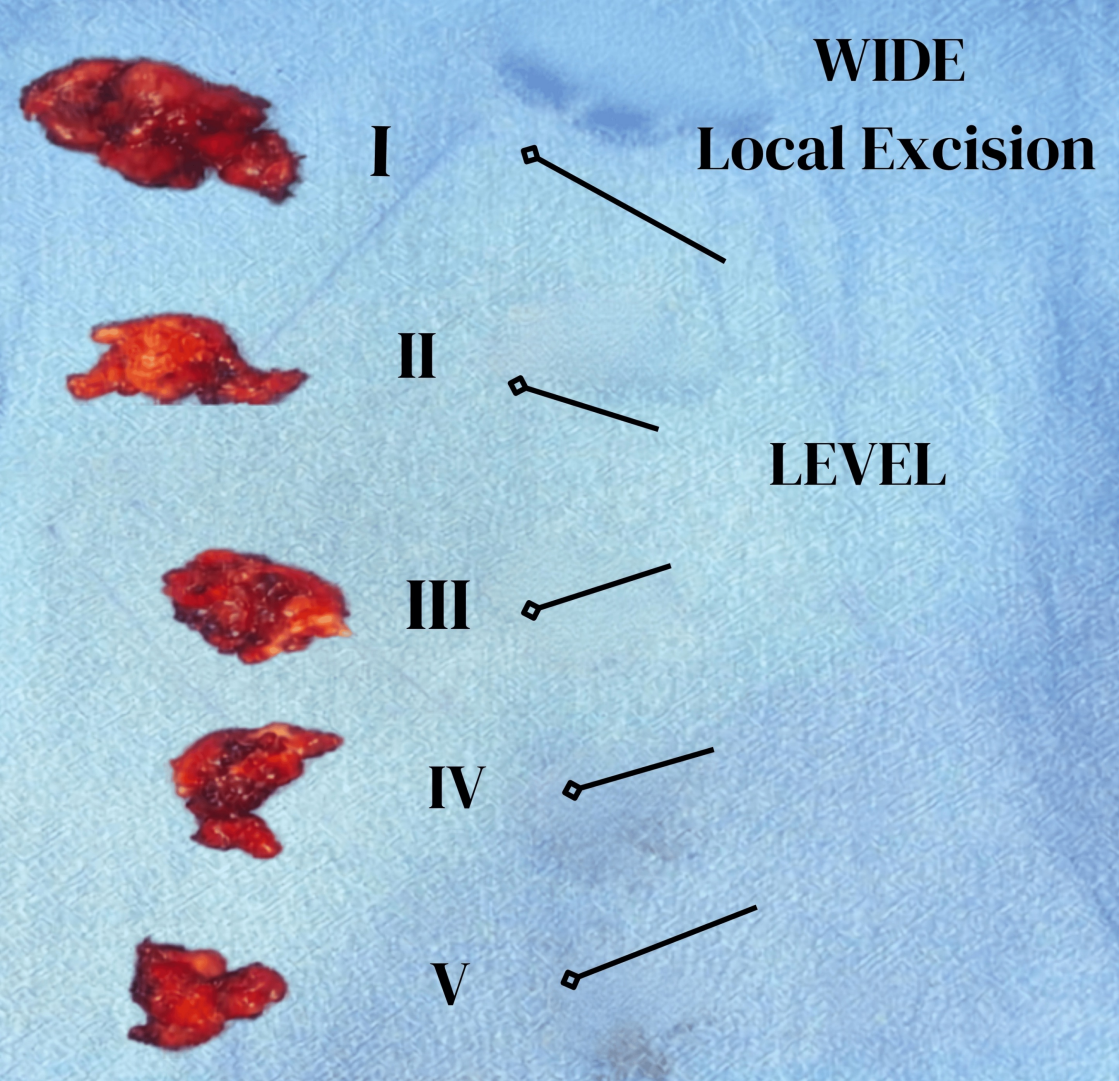
Lymph node dissection after lip carcinoma with nodal dissection of the neck

**Figure 3:**
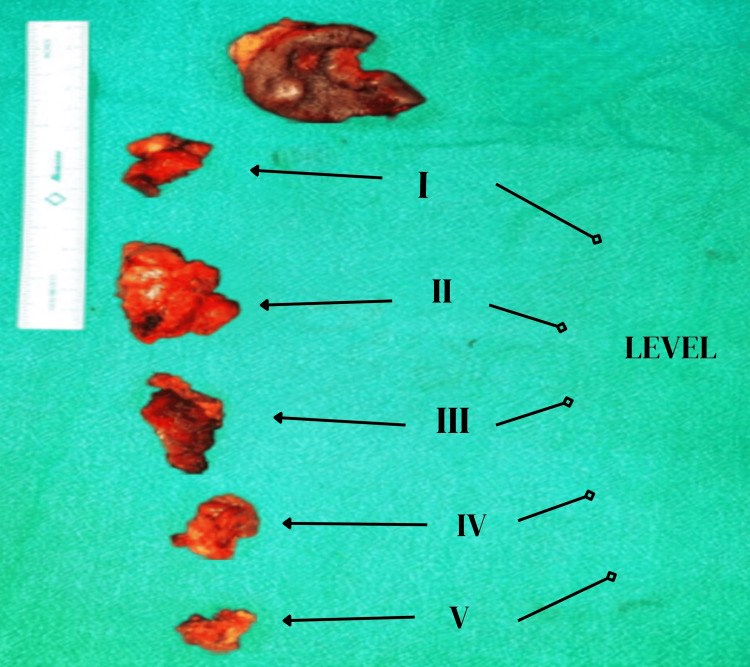
Wide local excision of the primary resection

The extent of neck dissection was determined according to the clinical stage and institutional surgical protocol. During surgery, level V lymph nodes were dissected separately from the remaining cervical lymph node specimen and submitted independently for histopathological examination. Representative operative specimens demonstrating wide local excision, composite resection, and level-wise cervical lymph node dissection with separate submission of nodal levels for histopathological examination are shown in Figures 1-3.

All surgical specimens were examined by experienced pathologists using standard histopathological criteria. Lymphovascular invasion (LVI) was defined as the presence of tumor cells within endothelial-lined lymphatic or vascular channels. Perineural invasion (PNI) was defined as tumor infiltration within, around, or through the nerve sheath. Extranodal extension (ENE) was defined as the extension of metastatic tumor beyond the lymph node capsule into the surrounding soft tissues. Histopathological examination was performed according to the institutional protocol, and all cervical nodal levels, including level V, were evaluated separately.

The clinical and pathological variables evaluated included tumor size, primary tumor site, clinical stage, pathological stage, histological differentiation, LVI, PNI, total number of lymph nodes harvested, number of metastatic lymph nodes, cervical nodal levels involved, and the presence of ENE. These variables were analyzed to determine their association with level V cervical lymph node metastasis.

Statistical analysis

Data were analyzed using SPSS (SPSS Statistics for Windows, version 17; SPSS Inc., Chicago, IL). Continuous variables were summarized using descriptive statistics, whereas categorical variables were expressed as frequencies and percentages. Associations between categorical variables were evaluated using the chi-square test or Fisher's exact test, as appropriate. Owing to the limited number of patients with level V lymph node metastasis (n = 7), multivariable regression analysis was not performed because the number of outcome events was insufficient to generate stable estimates. Therefore, the findings should be interpreted as univariate associations rather than independent predictors. A two-tailed p-value of <0.05 was considered statistically significant.

## Results

Table 1 summarizes the distribution of primary tumor sites and the overall level-wise pattern of cervical lymph node involvement among the 120 patients included in the study. Buccal mucosa/retromolar trigone was the most common primary site, while level IIA was the most frequently involved lymph node level.

**Table 1:** Distribution of primary tumor sites and level-wise pattern of lymph node involvement ENE = extranodal extension; LN = lymph node. Statistical analysis: Descriptive statistics

Variable	Category	N	%
Primary Tumor Site	Buccal mucosa/Retromolar trigone	52	43.3
Primary Tumor Site	Tongue/Floor of mouth	26	21.6
Primary Tumor Site	Gingivobuccal complex	28	23.3
Primary Tumor Site	Alveolar process	9	7.5
Primary Tumor Site	Lip	5	4.1
Primary Tumor Site	Total	120	100
Lymph Node Involvement	IA	2	1.6
Lymph Node Involvement	IB	29	24.16
Lymph Node Involvement	IIA	39	32.5
Lymph Node Involvement	IIB	9	7.5
Lymph Node Involvement	III	29	24.16
Lymph Node Involvement	IV	5	4.16
Lymph Node Involvement	V	7	5.83
Lymph Node Involvement	ENE	10	8.33

Table 2 presents the distribution of clinical and pathological nodal stages and their association with the primary tumor subsite. Tongue/floor of mouth tumors demonstrated more frequent involvement of levels II and III lymph nodes than other oral cavity subsites.

**Table 2:** Clinical and pathological nodal status according to oral subsite BM/RMT: buccal mucosa/retromolar trigone; GBS: gingivobuccal sulcus; FOM: floor of mouth; cN: clinical nodal stage; pN: pathological nodal stage. Statistical analysis: Descriptive statistics

Variable	Category	IA	IB	IIA	IIB	III	IV	V	pN0	pN1	pN2	pN3	Total
Clinical Stage	cN0	–	–	–	–	–	–	–	22	5	2	0	29
Clinical Stage	cN1	–	–	–	–	–	–	–	26	13	18	6	63
Clinical Stage	cN2	–	–	–	–	–	–	–	6	3	9	10	28
Clinical Stage	Total	–	–	–	–	–	–	–	54	21	29	16	120
Oral Subsite	Tongue/FOM	0	7	18	2	11	3	4	–	–	–	–	26
Oral Subsite	BM/RMT	1	12	10	4	8	0	1	–	–	–	–	52
Oral Subsite	GBS	0	8	4	2	5	2	2	–	–	–	–	28
Oral Subsite	Alveolus	0	3	5	1	5	0	0	–	–	–	–	9
Oral Subsite	Lip	1	3	2	0	0	0	0	–	–	–	–	5

Table 3 summarizes the distribution of pathological tumor (pT) stage and T4 category according to pathological nodal stage. Advanced pT stage and T4 lesions were more frequently observed among patients with advanced pathological nodal disease.

**Table 3:** Distribution of pathological tumor (pT) stage and T4 category according to pathological nodal stage pN: pathological nodal stage; LN: lymph node. Statistical analysis: Descriptive statistics

Variable	Category	IA	IB	IIA	IIB	III	IV	V	pN0	pN1	pN2	pN3	Total
Pathological Tumor Stage	pT1	0	2	8	0	2	0	0	–	–	–	–	12
Pathological Tumor Stage	pT2	0	9	8	2	11	0	0	–	–	–	–	30
Pathological Tumor Stage	pT3	1	13	11	3	5	2	1	–	–	–	–	36
Pathological Tumor Stage	pT4	1	5	12	4	11	3	6	–	–	–	–	42
T4 Category	Bone	–	–	–	–	–	–	–	3	0	14	7	24
T4 Category	Bone + Skin	–	–	–	–	–	–	–	0	0	3	5	8
T4 Category	Skin	–	–	–	–	–	–	–	0	0	1	0	1
T4 Category	>4 cm	–	–	–	–	–	–	–	0	0	6	3	9

The relationship of histopathological grade with PNI and LVI to pathological nodal metastasis can be seen in Table 4. Poorly differentiated tumors and tumors demonstrating PNI or LVI were more frequently observed among patients with advanced pathological nodal disease than among those without these features.

**Table 4:** Histopathological grade, PNI, and LVI in relation to nodal metastasis PNI, perineural invasion; LVI, lymphovascular invasion; pN, pathological nodal stage. Statistical analysis: Descriptive statistics

Variable	Category	pN0	pN1	pN2	pN3	Total
Histopathological Grade	Well differentiated	44	10	8	1	63
Histopathological Grade	Moderately differentiated	20	10	14	4	48
Histopathological Grade	Poorly differentiated	0	1	7	1	9
PNI	Positive	1	5	6	8	20
PNI	Negative	53	16	23	8	100
LVI	Positive	2	5	7	9	23
LVI	Negative	52	16	22	7	97
Total	–	54	21	29	16	120

Table 5 demonstrates a significant association between LVI, PNI, and pathological lymph node metastasis. Both LVI and PNI were significantly associated with nodal positivity (p<0.001).

**Table 5:** Correlation of LVI and PNI with lymph node metastasis LVI: lymphovascular invasion; PNI: perineural invasion; N(+): lymph node positive; N0: lymph node negative. Statistical analysis: Chi-square test/Fisher’s exact test. Statistical significance: p<0.05. *Statistically significant

Variable	N0	N(+)	Total	P-value
LVI Positive	2	21	23	0.0001*
LVI Negative	63	34	97	
PNI Positive	1	19	20	0.0001*
PNI Negative	64	36	100	
Total	65	55	120	

Table 6 summarizes the clinicopathological characteristics of patients with level V lymph node metastasis and the prevalence of level V involvement among clinically node-negative (cN0) patients. Level V metastasis was uncommon and was predominantly observed in patients with adverse pathological features, such as extracapsular spread and lymphovascular invasion.

**Table 6:** Characteristics of level V metastasis and prevalence of level V involvement in cN0 patients GBS: gingivobuccal sulcus; ENE: extranodal extension; LVI: lymphovascular invasion; pN: pathological nodal stage. Statistical analysis: Descriptive statistics

Variable	Category	pN0	pN1	pN2	pN3	ENE Positive	LVI Positive	Level V Positive	Total
Level V Metastasis Characteristics	Tongue	–	–	–	–	–	–	5	5
Level V Metastasis Characteristics	GBS	–	–	–	–	–	–	2	2
Level V Metastasis Characteristics	Male	–	–	–	–	–	–	6	6
Level V Metastasis Characteristics	Female	–	–	–	–	–	–	1	1
Level V Metastasis Characteristics	ENE Positive	–	–	–	–	6	–	7	7
Level V Metastasis Characteristics	LVI Positive	–	–	–	–	–	5	7	7
cN0 Patients	pN0	22	–	–	–	0	0	0	22
cN0 Patients	pN1	–	5	–	–	0	2	0	5
cN0 Patients	pN2	–	–	2	–	0	1	0	2
cN0 Patients	pN3	–	–	–	0	0	0	0	0
cN0 Patients	Total	22	5	2	0	1	3	0	29

Table 7 presents the prevalence of level V lymph node involvement among patients with cN1 disease. Patients presenting with clinically palpable level II/III lymph nodes demonstrated a higher frequency of level V metastasis than those with palpable level I nodes alone.

**Table 7:** Prevalence of level V lymph node involvement in cN1 patients cN: clinical nodal stage; pN: pathological nodal stage; ENE: extranodal extension; LVI: lymphovascular invasion; LN: lymph node. Statistical analysis: Descriptive statistics

Clinical Group	Category	pN0	pN1	pN2	pN3	ENE Positive	LVI Positive	Level V Positive	Total
cN1 (Level I)	pN0	23	–	–	–	0	0	0	23
cN1 (Level I)	pN1	–	9	–	–	0	4	0	9
cN1 (Level I)	pN2	–	–	16	–	0	4	0	16
cN1 (Level I)	pN3	–	–	–	4	2	1	1	4
cN1 (Level II/III)	pN0	3	–	–	–	0	0	0	3
cN1 (Level II/III)	pN1	–	4	–	–	0	2	0	4
cN1 (Level II/III)	pN2	–	–	2	–	0	1	0	2
cN1 (Level II/III)	pN3	–	–	–	4	4	1	1	4
Total cN1 (Level I)	All cases	23	9	16	4	2	9	1	52
Total cN1 (Level II/III)	All cases	3	4	2	4	4	4	1	11

Table 8 shows the prevalence of level V lymph node involvement among cN2 patients and across different clinical nodal stages. The frequency of level V metastasis increased with advancing nodal stage and was highest among patients with cN2 disease.

**Table 8:** Prevalence of level V lymph node involvement in cN2 patients and across clinical stages cN: clinical nodal stage; pN: pathological nodal stage; ENE: extranodal extension; LVI: lymphovascular invasion; LN: lymph node. Statistical analysis: Descriptive statistics

Variable	Category	pN0	pN1	pN2	pN3	ENE Positive	LVI Positive	Level V Positive	Total
cN2 Patients	pN0	6	–	–	–	0	0	0	6
cN2 Patients	pN1	–	3	–	–	0	2	0	3
cN2 Patients	pN2	–	–	9	–	0	5	2	9
cN2 Patients	pN3	–	–	–	10	6	4	3	10
Clinical Stage	cN0	–	–	–	–	–	–	0	29
Clinical Stage	cN1 (Level I)	–	–	–	–	–	–	1	52
Clinical Stage	cN1 (Level II/III)	–	–	–	–	–	–	1	11
Clinical Stage	cN2	–	–	–	–	–	–	5	28
Total	All Patients	6	3	9	10	6	11	7	120

Table 9 shows the association of level V lymph node metastasis to various clinicopathological factors. The factors of tumour size, histological differentiation, lymphovascular invasion, perineural invasion, tumour stage, and extracapsular extension were all shown to have a significant association with the level V nodal spread.

**Table 9:** Relationship between level V lymph node metastasis and clinicopathological factors BM/RMT: buccal mucosa/retromolar trigone; GBS: gingivobuccal sulcus; FOM: floor of mouth; LVI: lymphovascular invasion; PNI: perineural invasion; ENE: extranodal extension; LN: lymph node. Statistical analysis: Chi-square test and Fisher's exact test were used where appropriate. Statistical significance: p < 0.05 was considered statistically significant. Statistically significant

Variable	Category	Level V LN Positive (N)	Level V LN Negative (N)	Total (N)	Test Statistic	P-value
Gender	Male	6	79	85	0.79	0.30
Gender	Female	1	34	35	0.79	0.30
Age (years)	<40	1	31	32	1.10	0.57
Age (years)	40–60	5	75	80	1.10	0.57
Age (years)	>60	1	7	8	1.10	0.57
Tumor Size	cT1	0	40	40	8.10	0.04*
Tumor Size	cT2	3	50	53	8.10	0.04*
Tumor Size	cT3	2	23	25	8.10	0.04*
Tumor Size	cT4a	2	0	2	8.10	0.04*
Tumor Site	Tongue/FOM	4	22	26	5.90	0.20
Tumor Site	GBS	2	26	28	5.90	0.20
Tumor Site	Alveolus	0	9	9	5.90	0.20
Tumor Site	BM/RMT	1	51	52	5.90	0.20
Tumor Site	Lip	0	5	5	5.90	0.20
Differentiation	Well differentiated	1	62	63	14.50	0.0007*
Differentiation	Moderately differentiated	3	45	48	14.50	0.0007*
Differentiation	Poorly differentiated	3	6	9	14.50	0.0007*
LVI	Negative	2	95	97	13.10	0.0002*
LVI	Positive	5	18	23	13.10	0.0002*
PNI	Negative	1	99	100	25.50	<0.001*
PNI	Positive	6	14	20	25.50	<0.001*
Tumor Stage	I	0	11	11	12.50	0.005*
Tumor Stage	II	0	19	19	12.50	0.005*
Tumor Stage	III	1	63	64	12.50	0.005*
Tumor Stage	IV	6	20	26	12.50	0.005*
Extracapsular Extension	ENE Positive	6	4	10	58.20	<0.05*
Extracapsular Extension	ENE Negative	1	109	110	58.20	<0.05*
cT4 Category	Bone	2	22	24	7.80	<0.20
cT4 Category	Bone + Skin	1	7	8	7.80	<0.20
cT4 Category	Skin	0	1	1	7.80	<0.20
cT4 Category	>4 cm	4	5	9	7.80	<0.20

Table 10 examines the association between level V lymph node metastasis and involvement of level I and level II lymph nodes. While level I nodal status did not show a significant relationship, level II involvement demonstrated a statistically significant correlation with level V metastasis.

**Table 10:** Correlation of level V lymph node metastasis with level I and level II nodal status LN: lymph node. Statistical analysis: Chi-square test was used. Statistical significance: p<0.05 was considered statistically significant. *Statistically significant

Variable	Category	Level V LN Negative (N)	Level V LN Positive (N)	Chi-square	P-value
Level I Status	Negative	51	0	4.9	0.20
Level I Status	Positive	69	7	4.9	0.20
Level II Status	Negative	57	0	6.0	0.01*
Level II Status	Positive	63	7	6.0	0.01*

Table 11 assesses the relationship between level V lymph node metastasis and involvement of level III and level IV lymph nodes. Significant associations were observed between level III and level IV nodal involvement and level V metastasis.

**Table 11:** Correlation of level V lymph node metastasis with level III and level IV nodal status LN: lymph node. Statistical analysis: Chi-square test was used. Statistical significance: p<0.05 was considered statistically significant. *Statistically significant.

Variable	Category	Level V LN Negative (N)	Level V LN Positive (N)	Chi-square	P-value
Level III Status	Negative	78	0	11.7	0.0006*
Level III Status	Positive	42	7	11.7	0.0006*
Level IV Status	Negative	57	0	23.9	0.0001*
Level IV Status	Positive	63	7	23.9	0.0001*

Table 12 compares the level V nodal status with the neck disease stage. Although all level V-positive cases occurred in patients with advanced nodal disease (≥N2b), the association did not reach statistical significance.

**Table 12:** Comparison of level V nodal status with stage of neck disease N: number of patients; N2b: nodal stage according to the TNM classification; LN: lymph node. Statistical analysis: Chi-square test was used. Statistical significance: p < 0.05 was considered statistically significant.

Neck Disease Stage	Level V LN Negative	Level V LN Positive	Chi-square	P-value
N0-N2a	89	0	0.09	0.40
≥N2b	24	7	0.09	0.40

## Discussion

Cervical lymph node metastasis remains one of the most important prognostic indicators in patients with OSCC, significantly influencing disease staging, treatment planning, and survival outcomes. Although level V cervical lymph node metastasis is relatively uncommon, its presence has generally been associated with advanced disease and aggressive tumor behavior [12]. In the present study, advanced tumor stage, larger tumor size, poor histological differentiation, LVI, PNI, and ENE were significantly associated with pathological level V lymph node metastasis. These findings suggest that adverse clinicopathological characteristics frequently coexist in patients with advanced cervical nodal disease.

The present findings demonstrated that larger primary tumors and advanced pathological stage were more commonly observed among patients with level V lymph node metastasis. Increasing tumor burden is associated with greater local invasion and more extensive lymphatic dissemination, which may contribute to involvement of lower cervical nodal levels. Similar observations have been reported by Pandit et al., who identified LVI and PNI as clinicopathological factors associated with cervical lymph node metastasis [18], and by Alqutub et al., who emphasized the clinical importance of nodal metastasis and adverse pathological characteristics in patients with OSCC [19]. The findings of the present study are consistent with these reports and support the association between increasing tumor burden, aggressive pathological features, and more extensive cervical nodal involvement.

Adverse histopathological characteristics were also significantly associated with level V lymph node metastasis in the present study. Patients demonstrating LVI, PNI, poor histological differentiation, and ENE exhibited a higher frequency of level V involvement than patients without these pathological features. Comparable findings have been reported by Pandit et al., Alqutub et al., and Doll et al., who similarly observed that these pathological features are associated with more extensive cervical nodal disease [18-20]. Collectively, these observations suggest that adverse histopathological findings may assist clinicians in identifying patients who are more likely to have advanced cervical lymph node involvement.

Another important observation was the significant association between level V metastasis and pathological involvement of cervical lymph node levels II, III, and IV, whereas level I involvement was not significantly associated. This pattern is consistent with the recognized sequential spread of metastatic disease through the cervical lymphatic system. Doll et al. reported that, although cervical lymph node metastasis generally follows an orderly pattern, advanced tumors may demonstrate more extensive nodal dissemination involving lower cervical levels [20]. Similarly, Jayasuriya et al. reported an association between advanced primary tumors, higher nodal stage, and level V metastasis [21]. In the present study, all patients with level V metastasis had advanced pathological nodal disease (≥N2b); however, this association did not reach statistical significance, possibly because only seven patients demonstrated level V involvement.

Level V lymph node metastasis was identified in only 5.83% of the study population. Nevertheless, most affected patients demonstrated multiple adverse clinicopathological characteristics, including advanced tumor stage, poor histological differentiation, LVI, PNI, ENE, and extensive cervical nodal involvement. These findings suggest that assessment of these clinicopathological characteristics may be helpful during preoperative evaluation and surgical planning. However, because this study was observational and only a small number of patients demonstrated level V metastasis, the findings should be interpreted as associations rather than evidence of independent predictive factors. Similar observations have been reported by Jayasuriya et al. and Sahoo et al. [21,22].

Overall, the findings of the present study support the existing literature demonstrating that advanced tumor stage, adverse histopathological characteristics, and more extensive cervical nodal disease are associated with pathological level V lymph node metastasis in patients with OSCC. Recognition of these clinicopathological associations may assist surgeons during preoperative assessment and operative planning; however, larger multicenter studies are required before these factors can be considered independent predictors of level V metastasis.

Strengths and limitations

The present study has several strengths. Its prospective observational design, standardized surgical management, and separate histopathological evaluation of all cervical lymph node levels, including level V, enabled a comprehensive assessment of clinicopathological factors associated with pathological level V metastasis. In addition, multiple established pathological characteristics were evaluated simultaneously, providing clinically relevant information regarding the pattern of cervical lymph node involvement in patients with OSCC.

However, several limitations should be acknowledged. First, this was a single-center study with a relatively modest sample size. Second, only seven patients demonstrated level V lymph node metastasis, limiting the statistical power for subgroup analyses and precluding multivariable regression analysis to identify independent factors associated with level V involvement. Third, the observational design does not permit causal inference, and the findings should therefore be interpreted as associations rather than predictors. Finally, the results may not be generalizable to other institutions or patient populations. Future multicenter studies with larger sample sizes are warranted to validate these findings and determine the independent clinicopathological factors associated with level V lymph node metastasis in patients with OSCC.

## Conclusions

Level V cervical lymph node metastasis was uncommon in the present study but was predominantly observed in patients with advanced clinicopathological disease characteristics. Larger tumor size, advanced tumor stage, poor histological differentiation, LVI, PNI, ENE, and involvement of lower cervical lymph node levels (levels III and IV) were significantly associated with pathological level V lymph node metastasis. Recognition of these clinicopathological characteristics may assist clinicians in identifying patients who are more likely to demonstrate level V nodal involvement and may support individualized preoperative assessment and surgical planning. However, given the observational design and the limited number of patients with level V metastasis, these findings should be interpreted as associations rather than independent predictive factors. Further multicenter studies with larger sample sizes are warranted to validate these findings and better define clinicopathological factors associated with level V lymph node metastasis in patients with OSCC.
